# Escherichia coli Resistance to Fluoroquinolones in Community-Acquired Uncomplicated Urinary Tract Infection in Women: a Systematic Review

**DOI:** 10.1128/AAC.00862-20

**Published:** 2020-09-21

**Authors:** Ann E. Stapleton, Florian M. E. Wagenlehner, Aruni Mulgirigama, Monique Twynholm

**Affiliations:** a Department of Medicine, Division of Allergy and Infectious Disease, University of Washington, Seattle, Washington, USA; b Department of Urology, Pediatric Urology and Andrology, Justus Liebig University, Giessen, Germany; c GSK Global Specialty and Primary Care, London, United Kingdom; d GSK Medicines Research Centre, Stevenage, United Kingdom

**Keywords:** *Escherichia coli*, antimicrobial resistance, fluoroquinolone, urinary tract infection

## Abstract

Antibiotic resistance is a threat to public health, and uncomplicated urinary tract infections (uUTIs) are an example of this concern. This systematic review (International Prospective Register of Systematic Reviews [PROSPERO] ID: CRD42020156674) is the first to determine the prevalence of Escherichia coli resistance to fluoroquinolones in women with community-acquired uUTI. PubMed and Embase searches were conducted; 38 studies fulfilled eligibility criteria and were included in the systematic review. Within Europe, ciprofloxacin resistance in E. coli isolates varied between countries and increased in some from 2006 to 2008 and 2014 to 2016, specifically in the United Kingdom (0.5% to 15.3%), Germany (8.7% to 15.1%), and Spain (22.9% to 30.8%), although methodologies and settings were often not comparable. In Asia, there was a substantial increase in ciprofloxacin resistance during 2008 to 2014 from 25% to more than 40%. In North America, resistance to ciprofloxacin also increased between 2008 and 2017, from 4% to 12%. Data exploring different age groups did not show a consistent relationship with resistance, whereas two studies found that fluoroquinolone resistance was higher in postmenopausal women than premenopausal women. One study indicated a link between fluoroquinolone resistance and uUTI recurrence. These findings may have implications for the empirical treatment of uUTI with fluoroquinolones globally, but more data are needed to fully understand regional situations and impact patient management.

## Abstract (precis)

Antibiotic resistance is a threat to public health, and uncomplicated urinary tract infections (uUTIs) are an example of this concern. This systematic review (International Prospective Register of Systematic Reviews [PROSPERO] ID: CRD42020156674) is the first to determine the prevalence of Escherichia coli resistance to fluoroquinolones in women with community-acquired uUTI. PubMed and Embase searches were conducted; 38 studies fulfilled eligibility criteria and were included in the systematic review.

## INTRODUCTION

Community-acquired uncomplicated urinary tract infections (uUTIs) affect approximately 12% of women annually (1). In most cases, Escherichia coli is the causative pathogen (2–6), and antimicrobial resistance in this organism is becoming increasingly common worldwide (2, 5, 7).

Treatment of community-acquired uUTI remains largely empirical, and diagnosis is made based on the presence of characteristic lower urinary tract symptoms (8–11). With respect to specific antimicrobial therapies, US and international guidelines recommend first-line treatment with nitrofurantoin, trimethoprim-sulfamethoxazole (TMS) (according to local E. coli resistance patterns), fosfomycin, or pivmecillinam (8, 9). Recommendations regarding fluoroquinolones vary between guidelines. In Asia, these agents are currently recommended as a first-line treatment option for uUTIs due to the high frequency of TMS resistance in the region (10, 11). However, reflecting the globally rising rates of fluoroquinolone-resistant E. coli, US and European guidelines strongly recommend that fluoroquinolones are reserved for the treatment of uUTI only when there is no other alternative (8, 9). The evolving negative risk-benefit profile of fluoroquinolones (serious side effects and impact on the gut microbiome) has further restricted their use in these regions. The US Food and Drug Administration introduced black box warnings in 2016 and 2018, stating that fluoroquinolones should not be used in patients with uUTI unless no other treatment options are available, due to the risk of serious adverse effects (including effects on mental health, nervous system effects, and serious hypoglycemia) outweighing likely benefits (12, 13); the European Medicines Agency published a similar recommendation in March 2019 (14).

Despite this, several recent studies in the United States and Europe have shown that fluoroquinolones continue to be prescribed inappropriately for patients with uUTIs (15–18). A key concern is the suggestion that higher prescribing rates of certain antimicrobials (including penicillins, fluoroquinolones, nitrofurantoin, and trimethoprim) are directly associated with increased resistance of E. coli urinary tract isolates (19, 20). An association between extended-spectrum cephalosporin resistance in *Enterobacteriaceae* isolates from community-acquired UTIs and an increased risk of recurrent UTIs has also been reported (21).

To date, only two systematic reviews of fluoroquinolone resistance in UTIs caused by E. coli have been published; the first review is regarding ciprofloxacin resistance in men and women with community- and hospital-acquired UTIs (22), and the second relating to resistance in Korean women with uUTIs (23). No systematic reviews of the prevalence of E. coli resistance to fluoroquinolones specifically in women with community-acquired uUTIs have been conducted to date. As uUTIs are very common, it is timely to examine the epidemiology of fluoroquinolone resistance in order to inform regional practices regarding antimicrobial treatment. Therefore, the aim of this systematic review was to provide insights into the evolving epidemiology of antimicrobial resistance to fluoroquinolones in women with community-acquired uUTI caused by E. coli, with respect to variations over time, geography, and age.

## RESULTS

**FIG 1:**
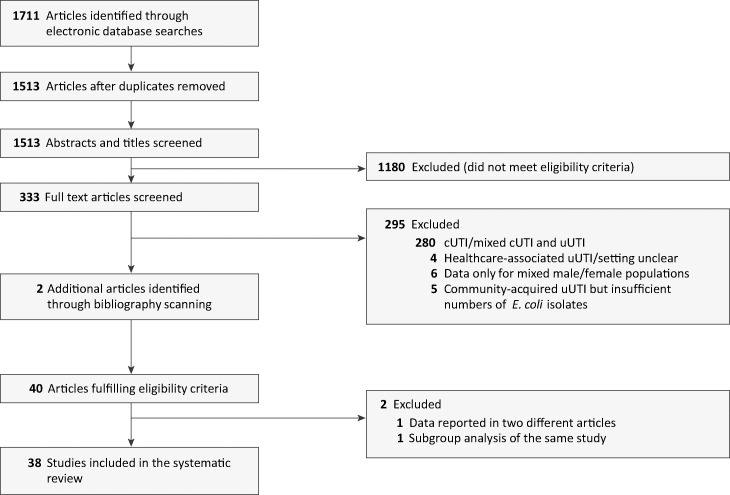
PRISMA flow diagram of study selection. cUTI, complicated urinary tract infection; uUTI, uncomplicated urinary tract infection.

A total of 1,711 articles were identified from the database searches (Fig. 1) and 1,513 remained after removal of duplicates. After a review of titles and abstracts, 333 articles were selected for review of the full text, and during this process, 2 other articles were identified from bibliographies (24, 25). Following examination of the full text, there were 40 articles reporting data from 38 studies that met the eligibility criteria (results of 1 study were published in 2 different journals, and a subgroup analysis of 1 study was published separately from the primary findings). Most articles were excluded for a lack of clarity regarding the study population (mixed male and female patients, whether the infection was community acquired or was specifically uUTI).

A summary of the included studies is presented in Table 1, with full details presented in Table S1 in the supplemental material. Most of the studies were conducted in Europe (*n* = 16), Asia (*n* = 12), and North America (*n* = 6), with 2 from South America, and 1 each from Saudi Arabia and Australia; none were found for Africa, as those published did not fulfil the eligibility criteria. Several studies reported susceptibility or resistance data, only a few studies reported both, and some studies classified intermediate susceptibility with either susceptible or resistant isolates. It was assumed that where these reports were unclear and only susceptibility data were presented, the remainder of the isolates tested were resistant. Most of the studies were of good quality, although many did not list limitations (see Table S2 in the supplemental material), and definitions of uUTI differed between reports (see Table S3 in the supplemental material).

**TABLE 1:** Characteristics of included studies according to geographical region^*a*^

Country by region (ref)	Setting	Reporting period	Age group (yrs)	E. coli isolate susceptibility to listed FQ(s) (%)	E. coli isolate resistance to listed FQ(s) (%)
Europe					
Austria (27)	PC/OPD	Jun 2007–Nov 2008	18–65	NR	CIP: 4.1
Denmark (67)	PC	Dec 2014–Dec 2015	18–65	NR	CIP: 8
France (26)	PC/OPD	2003–2006	18–65	CIP: 98.4	CIP: 1.4
France (70)	PC	2009–2011	18–65	LEV: 97, OFL: 97	NR
France (28)	PC	2014	≥18	CIP: 95.2	NR
Germany (29)	PC	Fall 2011	≥18	CIP: 91.3	CIP: 8.7
Germany (30)	OPD	Jan 2015–Jan 2017	≥18	CIP: 84.9, LEV: 86.3, MOX: 86.0	NR
Greece (71)	OPD	Jan 2005–March 2006	>16	NR	CIP: 2.2
Greece (72)	OPD	Jan 2005–Mar 2007	≥16	NR	CIP: 1.7
Greece (27)	PC/OPD	June 2007–Nov 2008	18–65	NR	CIP: 5.7
the Netherlands (4)	PC	Jan 2009–July 2009	≥11	CIP: 97, NOR: 97	NR
the Netherlands (31)	PC	Jan 2014–Jan 2015	≥11	CIP: 94	NR
Poland (33)	OPD	Mar–May 2013	19–94	CIP: 75.9	NR
Portugal (27)	PC/OPD	June 2007–Nov 2008	18–65	NR	CIP: 7.6
Spain (24)	OPD	June 2008–Mar 2009	Adult women	NR	CIP: 22.9, LEV: 22.5, NOR: 23.3
Spain (28)	PC	2014	≥18	CIP: 69.2	CIP: 30.8
Sweden (27)	PC/OPD	June 2007–Nov 2008	18–65	NR	CIP: 2.5
Sweden (28)	PC	2014	≥18	CIP: 92.7	CIP: 7.3
Sweden (32)	PC	Nov 2014–Mar 2016	≥17	NR	CIP: 1.1
Switzerland (73)	PC	Jun 2017–Aug 2018	≥18	CIP: 89.1, LEV: 86.5	NR
UK (27)	PC/OPD	June 2007–Nov 2008	18–65	NR	CIP: 0.5
UK (28)	PC	2014	≥18	CIP: 84.7	CIP: 15.3
North America					
Canada (36)	ED	2008	≥19	CIP: 90.5	CIP: 8.9
Canada (2)	PC	Apr 2009–Mar 2011	≥16	NR	CIP: 4.4
Canada (25)	ED/OPD	Apr 2010–Mar 2015	18–65	CIP; 2010: 92.1; 2011: 92.5; 2012: 91.4; 2013: 90.7; 2014: 90.3	NR
United States (38)	PC	Jan 2005–Dec 2007	College students	NR	CIP: 6.8
United States (35)	OPD	2005–2007	18-40	NR	CIP: 2.8, LEV: 2.8
United States (37)	ED	Sep 2016–Feb 2017	≥14	LEV: 88	NR
South America					
Brazil (6)	PC/OPD	Jan 2007–Jan 2009	≥14	NR	CIP: 9.2, LEV: 18.0, NOR: 7.4
Asia Pacific					
Australia (49)	PC/OPD	Jun 2009–Jul 2011	15–45	NR	CIP: 13, NOR: 12
China (43)	PC	Jan 2012–Dec 2013	≥16	CIP: 76.6	CIP: 23.4
Hong Kong (44)	PC/ED/ OPD	Jan 2006–June 2008	≥18	CIP: 87.1	CIP: 12.9
Japan (39)	OPD	Jan–Dec 2008	Adults	CIP: 91.8, LEV: 91.8, SIT: 98.0, TOS: 91.5	NR
Japan (40)	OPD	2007–2009	NR	LEV: 85.8	NR
Japan (41)	OPD	Aug 2015–May 2017	16–90	LEV: 84.6	LEV: 15.4
Korea (42)	OPD	May–Oct 2006	18–65	NR	CIP: 26.7
Korea (3)	OPD	Jan 2008–June 2009	Adults	NR	CIP: 24.8, LEV: 21.3
Korea (23)	OPD	Jan 2010–Dec 2014	Adults	CIP: 58.3, LEV: 61.3	NR
Pakistan (45)	OPD	Dec 2011–June 2012	≥18	CIP: 67.2	CIP: 32.8
Philippines (74)	ED/OPD	July 2010–Oct 2011	≥18	NR	LEV: 5.7
Turkey (46)	ED/OPD	Mar 2005–Sep 2006	18–65	NR	All FQs: 25.2
Turkey (47)	OPD	Jan–Dec 2007	18–65	NR	CIP: 22
Middle East					
Saudi Arabia (48)	PC/OPD	May 2015–Apr 2016	≥18	NR	CIP: 25.4

a Where available, data for individual countries from the same study are listed separately. Note: ECO·SENS 2014 update data from Germany have not been included, as it could not be confirmed that data were exclusively from community-acquired uUTIs. Data from some individual European countries in the ARESC study have not been included where fewer than 100 E. coli isolates were analyzed. CIP, ciprofloxacin; ED, emergency department; FQ, fluoroquinolones; LEV, levofloxacin; NOR, norfloxacin; NR, not reported; OFL, ofloxacin; OPD, outpatients department; PC, primary care; ref, reference; SIT, sitafloxacin; TOS, tosufloxacin; uUTI, uncomplicated urinary tract infection.

Results of studies conducted in primary care, emergency department, and outpatient settings were reported for multiple countries across Europe, with some articles presenting findings from several countries as part of larger projects (Table 1). In particular, Antimicrobial Resistance Epidemiological Survey on Cystitis (ARESC) was conducted in primary care and outpatient settings and captured data from nine European countries (2003 to 2006), with reported ciprofloxacin resistance rates of between 1.4% for France and 12.9% for Russia (26). The ECO·SENS studies also provided data across several countries within Europe within primary care and outpatient urology clinics for women aged 18 to 65 years at two time points, namely, 2008 and 2014 (27, 28). In ECO·SENS II, in 2008 rates of resistance to ciprofloxacin ranged from 0% in Sweden to 5.8% in Portugal. This finding compares with 2014 data from the ECO·SENS update of 4.8% for France and 30.8% for Spain. However, there were some differences in the countries included (Portugal, Greece, and Austria were only included in 2008; while France, Germany, and Spain were only included in 2014). Also, ECO·SENS II reported data from both primary care and outpatient settings, whereas the 2014 update reported data from only primary care (27, 28).

Many articles provided data allowing insight into changes in the prevalence of E. coli fluoroquinolone resistance for individual countries within Europe, and some articles indicated an increase over a short period of time. In the United Kingdom, resistance to ciprofloxacin was reported as 0.5% in 2008, sharply rising to 15.3% in 2014 in ECO·SENS II and the ECO·SENS 2014 update, respectively (27, 28). However, it should be noted that data were provided by 12 centers in the United Kingdom for ECO·SENS II and only 2 centers in the ECO SENS 2014 update, which could impact these findings. In Germany, a primary care study reported a ciprofloxacin resistance rate of 8.7% in 2011 (29), with a higher incidence of 15.7% in a separate, later outpatient study (30), and a similar picture was reported for the Netherlands (from 3% in 2009% to 6% in 2014), with both studies using the same nationwide primary care data source (4, 31). Resistance rates in Spain were somewhat higher than in other European countries, and data from a large study (2008 to 2009) showed a ciprofloxacin resistance rate of 22.9% compared with 30.8% in ECO·SENS (2014); albeit, the latter rate was from a more geographically restricted sample (24, 28). Data from primary care in Sweden are more difficult to interpret; in ECO·SENS II (2008), resistance to ciprofloxacin was 2.5% (27), increasing to 7.3% in the ECO·SENS 2014 update (28), although another separate study reported a ciprofloxacin resistance rate of only 1.1% in 2016 (32). However, these studies sampled populations with different demographics. Notably, when 2009 data from the Netherlands were categorized according to age, resistance to ciprofloxacin was lowest for patients aged 21 to 50 years (1%) and highest for those aged 51 to 70 years (5%) (Table 2) (4). A study from Poland reported a greater discrepancy in ciprofloxacin resistance according to age—in patients younger than 65 years, resistance was 11.1%, but this value rose to 45.4% in those aged 65 years and above (33). A similar pattern was observed in Belgium, but the differences in resistance according to age (18 to 55 years versus >55 years) were much less pronounced (4.2% and 9.4%, respectively) (34).

**TABLE 2:** Studies reporting fluoroquinolone resistance data^*a*^

Country or region by reporting category (ref)	Setting	Reporting period	E. coli isolate susceptibility to listed FQ(s) by category (%)	E. coli isolate resistance to listed FQ(s) by category (%)
Age				
Belgium (34)	PC	May 2014–Dec 2015	LEV/OFL: 18–55 years, 95.8; >55 years, 90.6	NR
the Netherlands (4)	PC	Jan 2009–July 2009	CIP: 11–20 years, 98; 21–50 years, 99; 51–70 years, 95; >70 years, 97 NOR: 11–20 years, 97; 21–50 years, 98; 51–70 years, 95; >70 years, 97	NR
the Netherlands (31)	PC	Jan 2014–Jan 2015	CIP: 11–20 years, 93; 21–50 years, 96; 51–70 years, 97	NR
Poland (33)	OPD	Mar–May 2013	CIP: <65 years, 88.9; ≥65 years, 54.6	NR
Japan (41)	OPD	Aug 2015–May 2017	LEV: <65 years, 83.3; ≥65 years, 85.2	LEV: <65 years, 16.7; ≥65 years, 14.8
Pakistan (45)	OPD	Dec 2011–June 2012	CIP: 18–30 years, 89.7; 31–40 years, 64.3; 41–50 years, 58.1; 51–60 years, 58.1; 61–75 years, 61.5	CIP: 18–30 years, 10.3; 31–40 years, 35.7; 41–50 years, 41.9; 51–60 years, 41.9; 61–75 years: 38.5
Turkey (46)	ED/OPD	Mar 2005–Sep 2006	NR	FQs: <50 years, 22.7; ≥50 years, 31.3
Menopausal status				
Poland (50)	OPD	2013–2015	NR	CIP: premenopausal, 10.7; postmenopausal, 22.7
Japan (51)	OPD	Jan–Dec 2008	CIP: premenopausal, 94.4; postmenopausal, 89.4 LEV: premenopausal, 94.4; postmenopausal, 89.5 SIT: premenopausal, 99.3; postmenopausal, 96.5 TOS: premenopausal, 93.9; postmenopausal, 89.1	NR
Recurrent uUTI				
9 European countries and Brazil (26)	PC/OPD	2003–2006	CIP: nonrecurrent, 92.8; recurrent, 83.0	CIP: nonrecurrent, 7.0; recurrent, 17.0

a CIP, ciprofloxacin; ED, emergency department; FQ, fluoroquinolones; LEV, levofloxacin; NOR, norfloxacin; NR, not reported; OFL, ofloxacin; OPD, outpatients department; PC, primary care; SIT, sitafloxacin; TOS, tosufloxacin; uUTI, uncomplicated urinary tract infection.

Six articles concerned studies conducted in North America (2, 25, 35–38), and one in particular from Canada allows an insight into changing resistance to ciprofloxacin in this region. In the report by Delisle et al. of data from the same primary care, emergency department, and outpatient settings tracked ciprofloxacin resistance from 2010 to 2014 and showed only a slight increase in resistance from 7.9% to 9.7% (although the authors stated that this was a statistically significant change) (25). In emergency department data from Canada in 2008, resistance to ciprofloxacin was reported in 8.9% of E. coli isolates tested (36), compared with 2.8% in a separate US outpatient study (2005 to 2007) (35). In comparison, a more recent emergency department study conducted in the United States reported resistance to levofloxacin of 12% between 2016 and 2017 (37).

Most data from Asia were reported for the Eastern region. In three studies from Japan, reported rates of E. coli resistance to levofloxacin were 8.2% (nationwide), 14.2% (single center), and 15.4% (single center) in 2008, 2009, and 2017, respectively (39–41). In the latest data set, E. coli levofloxacin resistance was 16.7% in women aged less than 65 years compared with 14.8% for those aged 65 years and above in the outpatient setting (Table 2) (41). Data from the outpatient setting in Korea also permit the tracking of resistance trends in Eastern Asia. In 2006, resistance to ciprofloxacin was reported as 26.7% (42), falling slightly to 24.8% in 2009 (3) but then increasing significantly, as reported in one study, to 41.7% during 2010 to 2014 (23). However, while the latter was a single center study, the previous two were nationwide samples. A single primary care study from Southern China reported a resistance to ciprofloxacin of 23.4% in isolates collected between 2012 and 2013 (43). Notably, an earlier study from Hong Kong conducted during 2006 to 2008 reported a much lower resistance rate to ciprofloxacin (12.9%) (44).

A single article has provided ciprofloxacin resistance data from Southern Asia in an outpatient setting in Pakistan, with the 2012 data analyzed according to age (45). A steady increase in resistance with older age was observed, from 10.3% at age 18 to 30 years to 41.9% at age 51 to 60 years, with a decline in patients aged over 60 years (38.5%). Two separate studies from Turkey have reported resistance rates higher than 20% for all fluoroquinolones, again showing a relationship between older age and increased resistance (46, 47).

Only single studies have been published from some other regions. Araujo et al. reported resistance data from the primary care and outpatient settings in Brazil, with isolates collected between 2007 and 2009 (6). Reported resistance to ciprofloxacin and norfloxacin was 9.2% and 7.4%, respectively, but higher to levofloxacin (18%), which is notable, when these values might be expected to be similar. During a similar time period, a separate study reported a ciprofloxacin resistance rate of 10.8% in E. coli isolates from Brazil (26). The only article reporting data for the Middle East region was a recent publication from Saudi Arabia (48). Isolates from primary care and outpatient departments were resistant to ciprofloxacin in 25.4% of cases. A single study conducted in Australia between 2009 and 2011 reported resistance to ciprofloxacin of 13% (49).

Differences in resistance rates according to menopausal status were examined in two separate studies (Table 2). In Poland, resistance to ciprofloxacin in premenopausal women was reported as 10.8% versus 24.2% in postmenopausal women (50). In an earlier Japanese study, ciprofloxacin resistance was 6.9% for premenopausal women and 12.6% for postmenopausal women (51).

Only the ARESC study presented information with respect to uUTI recurrence (Table 2). Data collected from 2003 to 2006 from nine European countries and Brazil reported a resistance rate to ciprofloxacin in 7% of women with nonrecurrent uUTI with an increase to 17% in those with recurrent uUTI (26). However, in the study conducted by Kim et al. in Korea, testing isolates from 2010 to 2014, resistance to ciprofloxacin was 68.7% in women whose present episode had not resolved with antibiotic treatment within the previous 3 months, compared with 35.8% of women with no previous antibiotic exposure for their uUTIs (23). Notably, the authors found no evidence of a relationship between recurrence and antibiotic resistance.

## DISCUSSION

The aim of this systematic review was to determine the prevalence of fluoroquinolone resistance in women with community-acquired uUTIs caused by E. coli across different geographical regions and care settings, which might inform the development of local guidelines for antibiotic prescribing. Resistance to fluoroquinolones was most common in Asia (particularly China and Korea) where data from separate studies show a substantial increase in resistance to ciprofloxacin between 2008 and 2014 (3, 23, 39–45). A few articles from studies using similar methodologies provided evidence of how rates of resistance to fluoroquinolones have changed over time in Europe (4, 24, 27–34) and North America (2, 25, 35–38), and they report clear differences from those in Asia with respect to absolute percentages of resistant isolates and also the magnitude of change over time.

The recommendations in Japanese and Korean guidelines to use fluoroquinolones as an empirical treatment option for uUTI due to the high frequency of resistance to TMS in the region might contribute to the development of resistance in these regions (10, 11). However, it must be noted that a high volume of fluoroquinolone prescribing for other conditions (52) is likely to have a greater impact on fluoroquinolone resistance both in the individual and the community. Indeed, it has been shown that high fluoroquinolone use in general has been associated with an increased prevalence of resistance in E. coli over time in Eastern Asia (53–56).

Conversely, fluoroquinolones are not recommended in European and US guidelines unless no other alternatives are available, reflecting the growing prevalence of resistance cited in the present review (8, 9). However, fluoroquinolones continue to be prescribed for patients with uUTIs, which conflicts with regulatory and guideline recommendations in these regions. In an analysis of commercial insurance data in the United States, it was found that fluoroquinolones were prescribed in outpatient and emergency department settings for more than 40% of cases of uUTI in women aged younger than 45 during 2009 to 2013 (15), which is a slightly higher percentage than that reported in another national outpatient survey of women from 2002 to 2011 (16). A separate primary care survey conducted in the Netherlands from 2007 to 2010 reported use of fluoroquinolones in more than 40% of uUTI patients (17).

In contrast, antimicrobial stewardship programs and education that restrict the use of fluoroquinolones for antibiotic resistant cases or more serious infections may result in decreased resistance rates in uUTIs. In a study conducted in the United States, an emergency department-specific antibiogram was developed to determine the rates of E. coli resistance to commonly used antibiotics (57). This information, together with the US guidelines was used to devise recommendations for the treatment of uUTIs within that institution, and these guidelines were disseminated through education delivered by pharmacists in the emergency department and during regular physician meetings. Before the educational intervention, the choice of empirical antibiotic therapy was consistent with recommendations in 49% of cases, and this value increased substantially to 83% posteducation, with the key changes being decreased prescribing of TMS and fluoroquinolones. Notably, the percentage of E. coli isolates from uUTIs that were susceptible to prescribed antibiotics rose from 74% to 89% (57).

One issue that may become more important over time is the prescription of antibiotics during non-face-to-face health care consultations. The challenge with this approach is that care provided by patient electronic record-based or telephone management does not provide the opportunity to obtain urine samples to confirm resistance, often lacks documentation of treatment strategies, and is not necessarily assigned to trained health care providers. In a US-based primary care study during 2015 to 2016, 34% of all prescriptions for antibiotics were issued without a face-to-face consultation (58). A separate large analysis of claims data in the United States has shown that although only 9% of consultations for UTIs were online, they resulted in an antibiotic prescription rate of 76%, which was higher than that for either primary care or emergency department visits (59). Another primary care study showed that while antibiotics were prescribed on 49% of face-to-face consultations for suspected UTIs, this value increased to 99% for online consultations (60). However, this conclusion does not necessarily mean that antibiotics were prescribed inappropriately in these settings. A related problem is that in numerous countries around the world, antibiotics are available without a prescription or are dispensed by pharmacies without a prescription (61, 62), and patients may also keep leftover antibiotics to self-treat a future uUTI (63, 64). It has yet to be established how these factors impact resistance development, and more research is needed.

Some studies in the systematic review shed light on how E. coli fluoroquinolone resistance rates vary with age or menopausal status, although findings are conflicting and differ according to geographical location. In one study conducted in Japan with data from 2008, there was a consistently higher resistance to fluoroquinolones in postmenopausal women relative to premenopausal women (51). A similar finding has been reported with respect to ciprofloxacin resistance in a large study from Poland (2013 to 2015) (50). This finding is of particular interest, as in some current guidelines, the criteria for uUTI exclude postmenopausal women, i.e., they are considered to have a complicated UTI (8, 9), which may provide some explanation. Notably, Japanese guidelines state that there is a high rate of E. coli resistance to fluoroquinolones in postmenopausal women with UTIs; consequently, penicillins or cephalosporins are the recommended first-line treatment options (10).

Only one article reported resistance rates in women with recurrent community-acquired uUTIs. This international study included patients from nine European countries and Brazil (2003 to 2006), which showed a 10% higher resistance rate in those with recurrent uUTIs (26). However, in a Korean study, although E. coli resistance was more frequent in women with unresolved uUTIs following antibiotic treatment, there was no apparent relationship between recurrence and antibiotic resistance (23). This study underlines the urgent need for more (recent) data in order to explore this finding further. In contrast to patients with a first presentation of uUTIs, for women with recurrent uUTIs, urine culture is recommended when feasible by current guidelines in advance of prescribing antimicrobial therapy, with a consideration of local resistance rates (antibiograms), in order to facilitate appropriate treatment selection and reduce antibiotic overuse (9, 10, 65). However, empirical treatment may be initiated by the patient themselves if urine culture is not possible, and it has been shown that many women with recurrent uUTIs can accurately self-diagnose and successfully treat their condition with antibiotics (66). Striking a balance between timely treatment of recurrent uUTIs and the risk of resistance due to regular antibiotic consumption is an important issue that may not be easily resolved.

There are a number of limitations to this systematic review. Chiefly, they are related to the various methodologies across studies (including the definitions of uUTI) that make it difficult for a direct comparison between geographical regions and to follow trends over time. The lack of studies for this specific population in Africa, Eastern Europe, and South America emphasize this issue. Much of the data within the articles were collected prior to 2008, making interpretations of the current status and trends over time of fluoroquinolone resistance difficult, which impacts the utility of the information with respect to developing local guidelines and informing clinical practice. However, a key strength of this systematic review was the stringent inclusion criteria, which eliminated many articles where eligibility of the study population was unclear.

This systematic review indicates that resistance to fluoroquinolones in women with community-acquired uUTI caused by E. coli is a growing concern, specifically in Asia but also in some countries within Europe; an upward trend in North America is also detectable. However, more contemporary data are needed from around the world to provide a greater insight into the precise nature of this problem. The primary goal of antibiotic treatment selection is to provide effective empirical therapy while avoiding selection for future resistance, by minimizing use of broad-spectrum agents. In cases of antibiotic resistant uUTI, treatment that is not informed by urine culture and susceptibility testing may have a negative impact on antimicrobial stewardship. A proactive approach for identifying predictors in women at risk of antibiotic-resistant uUTI is imperative to define the right management approach to improve treatment outcomes in the short and long term.

## MATERIALS AND METHODS

The protocol for this systematic review was developed according to Preferred Reporting Items for Systematic Review and Meta-Analysis Protocols (PRISMA-P) guidelines (68) and was registered on the International Prospective Register of Systematic Reviews (PROSPERO; ID: CRD42020156674; www.crd.york.ac.uk/prospero). The search strategy and study selection are summarized below, with full details in the supplemental material.

Embase and PubMed searches were conducted using keywords only and keywords with medical subject headings (MeSH) within the title, abstract, or full text of papers. Additionally, bibliographies from the papers identified during searches were also hand-searched. The databases were searched for English language articles published between 1 January 2009 and 2 December 2019.

Papers were included in the systematic review only if they were peer-reviewed publications reporting results of observational studies. Studies were required to include only nonpregnant females aged 12 years or above with community-acquired uUTIs (first presentation or recurrent). uUTI was defined as UTI in the absence of upper urinary tract infection, pyelonephritis, any relevant functional or anatomical abnormalities in the urinary system, or catheter-associated UTI. Community-acquired uUTI was defined as samples from patients presenting in outpatient clinics or primary care or at presentation to the emergency department, without prior hospitalization for the index episode, excluding patients in residential/long-term-care facilities. Within individual studies, patients with chronic medical conditions, e.g., diabetes and immunosuppression, were excluded. Other eligibility criteria included the documentation of validated methods of urine culture and susceptibility testing (European Committee on Antimicrobial Susceptibility Testing or Clinical and Laboratory Standards Institute) guidance relevant at the time of the study and the testing of at least 100 E. coli-positive isolates in each study. For articles where it was unclear that the data reported applied only to the population of interest, the authors were contacted to provide clarification, e.g., Ismail et al. (35).

Two independent reviewers (F.M.E.W. and A.E.S.) screened the database search results (one each for Embase and PubMed results). Results were first saved as Microsoft Word (v.16) files in order to facilitate screening of the studies identified. Titles and abstracts were screened to determine eligibility, followed by review of the full articles against the inclusion/exclusion criteria, including where there was uncertainty based on the abstract/title. Articles not meeting all inclusion criteria were excluded.

Data from full publications were extracted into Microsoft Excel (v.16) by F.M.E.W., and an independent random sample of data (10% of the total eligible papers identified) was extracted by A.E.S. to ensure agreement. Data extracted included bibliographic information, country/region of the study, study setting, time frame, patient demographics, resistance testing methods, and all outcomes regarding susceptibility testing/antimicrobial resistance. When only susceptibility data were reported, it was assumed that the remainder of isolates tested in a study were resistant, unless otherwise stated. Duplicate articles were removed. The quality of studies and reporting was assessed by the reviewers according to an abbreviated version of the Appraisal Tool for Cross-sectional Studies (AXIS) (69).

## NOTE ADDED AFTER PUBLICATION

This article was published on 21 September 2020. On 8 December 2021, the following changes were made.

References 38 to 73 in the References list have been renumbered, and a few additional corrections to the in-text citation numbers have been made.

## References

[1] Foxman B, Brown P. 2003. Epidemiology of urinary tract infections: transmission and risk factors, incidence, and costs. Infect Dis Clin North Am 17:227–241. doi:10.1016/S0891-5520(03)00005-9. 12848468

[2] McIsaac WJ, Moineddin R, Meaney C, Mazzulli T. 2013. Antibiotic-resistant *Escherichia coli* in women with acute cystitis in Canada. Can J Infect Dis Med Microbiol 24:143–149. doi:10.1155/2013/547848. 24421825 PMC3852451

[3] Lee SJ, Lee DS, Choe HS, Shim BS, Kim CS, Kim ME, Cho Y. 2011. Antimicrobial resistance in community-acquired urinary tract infections: results from the Korean Antimicrobial Resistance Monitoring System. J Infect Chemother 17:440–446. doi:10.1007/s10156-010-0178-x. 21140281

[4] den Heijer CDJ, Donker GA, Maes J, Stobberingh EE. 2010. Antibiotic susceptibility of unselected uropathogenic *Escherichia coli* from female Dutch general practice patients: a comparison of two surveys with a 5 year interval. J Antimicrob Chemother 65:2128–2133. doi:10.1093/jac/dkq286. 20682565

[5] Hayami H, Takahashi S, Ishikawa K, Yasuda M, Yamamoto S, Wada K, Kobayashi K, Hamasuna R, Minamitani S, Matsumoto T, Kiyota H, Tateda K, Sato J, Hanaki H, Masumori N, Nishiyama H, Miyazaki J, Fujimoto K, Tanaka K, Uehara S, Matsubara A, Ito K, Hayashi K, Kurimura Y, Ito S, Takeuchi T, Narita H, Izumitani M, Nishimura H, Kawahara M, Hara M, Hosobe T, Takashima K, Chokyu H, Matsumura M, Ihara H, Uno S, Monden K, Sumii T, Kawai S, Kariya S, Sato T, Yoshioka M, Kadena H, Matsushita S, Nishi S, Hosokawa Y, Shirane T, Yoh M, Watanabe S, Makinose S, Uemura T, Goto H. 2019. Second nationwide surveillance of bacterial pathogens in patients with acute uncomplicated cystitis conducted by Japanese Surveillance Committee from 2015 to 2016: antimicrobial susceptibility of *Escherichia coli*, *Klebsiella pneumoniae*, and *Staphylococcus saprophyticus*. J Infect Chemother 25:413–422. doi:10.1016/j.jiac.2019.02.021. 30905628

[6] Araújo SMHA, Mourão TC, Oliveira JL, Melo IFS, Araújo CAA, Araújo NAA, Melo MCA, Araújo SR, Daher EF. 2011. Antimicrobial resistance of uropathogens in women with acute uncomplicated cystitis from primary care settings. Int Urol Nephrol 43:461–466. doi:10.1007/s11255-010-9777-9. 20559725

[7] Butler CC, Francis N, Thomas-Jones E, Llor C, Bongard E, Moore M, Little P, Bates J, Lau M, Pickles T, Gal M, Wootton M, Kirby N, Gillespie D, Rumbsy K, Brugman C, Hood K, Verheij T. 2017. Variations in presentation, management and patient outcomes of urinary tract infection: a prospective four-country primary care observational cohort study. Br J Gen Pract 67:e830–e841. doi:10.3399/bjgp17X693641. 29158245 PMC5697553

[8] Gupta K, Hooton TM, Naber KG, Wullt B, Colgan R, Miller LG, Moran GJ, Nicolle LE, Raz R, Schaeffer AJ, Soper DE, European Society for Microbiology and Infectious Diseases. 2011. International clinical practice guidelines for the treatment of acute uncomplicated cystitis and pyelonephritis in women: a 2010 update by the Infectious Diseases Society of America and the European Society for Microbiology and Infectious Diseases. Clin Infect Dis 52:e103–e120. doi:10.1093/cid/ciq257. 21292654

[9] Bonkat G, Pickard R, Bartoletti R, Cai T, Bruyère F, Geerlings SE, Köves B, Wagenlehner F. 2019. EAU guidelines on urological infections. European Association of Urology, Arnhem, The Netherlands. https://uroweb.org/guideline/urological-infections/.

[10] The Japanese Association for Infectious Disease/Japanese Society of Chemotherapy, The JAID/JSC Guidelines to Clinical Management of Infectious Disease Preparing Committee, Urinary Tract Infection/Male Genital Infection Working Group, Yamamoto S, Ishikawa K, Hayami H, Nakamura T, Miyairi I, Hoshino T, Hasui M, Tanaka K, Kiyota H, Arakawa S. 2017. JAID/JSC guidelines for clinical management of infectious disease 2015—urinary tract infection/male genital. J Infect Chemother 23:733–751. doi:10.1016/j.jiac.2017.02.002. 28923302

[11] Kang CI, Kim J, Park DW, Kim B, Ha U, Lee S, Yeo JK, Min SK, Lee H, Wie S. 2018. Clinical practice guidelines for the antibiotic treatment of community-acquired urinary tract infections. Infect Chemother 50:67–100. doi:10.3947/ic.2018.50.1.67. 29637759 PMC5895837

[12] US Food and Drug Administration. 2016. FDA drug safety communication: FDA updates warnings for oral and injectable fluoroquinolone antibiotics due to disabling side effects. US Food and Drug Administration, Harrisburg, PA. https://www.fda.gov/media/99425/download.

[13] US Food and Drug Administration. 2018. FDA updated warnings for fluoroquinolone antibiotics on risks of mental health and low blood sugar adverse reactions. US Food and Drug Administration, Harrisburg, PA. https://www.fda.gov/news-events/press-announcements/fda-updates-warnings-fluoroquinolone-antibiotics-risks-mental-health-and-low-blood-sugar-adverse.

[14] European Medicines Association. 2019. Disabling and potentially permanent side effects lead to suspension or restrictions of quinolone of fluoroquinolone antibiotics. European Medicines Association, Amsterdam, The Netherlands. https://www.ema.europa.eu/en/documents/referral/quinolone-fluoroquinolone-article-31-referral-disabling-potentially-permanent-side-effects-lead_en.pdf.

[15] Durkin MJ, Keller M, Butler AM, Kwon JH, Dubberke ER, Miller AC, Polgreen PM, Olsen MA. 2018. An assessment of inappropriate antibiotic use and guideline adherence for uncomplicated urinary tract infections. Open Forum Infect Dis 5:ofy198. doi:10.1093/ofid/ofy198. 30191156 PMC6121225

[16] Kobayashi M, Shapiro DJ, Hersh AL, Sanchez GV, Hicks LA. 2016. Outpatient antibiotic prescribing practices for uncomplicated urinary tract infection in women in the United States, 2002–2011. Open Forum Infect Dis 3:ofw159. doi:10.1093/ofid/ofw159. 27704014 PMC5047404

[17] van den Broek d'Obrenan J, Verheij TJM, Numans ME, van der Velden AW. 2014. Antibiotic use in Dutch primary care: relation between diagnosis, consultation and treatment. J Antimicrob Chemother 69:1701–1707. doi:10.1093/jac/dku005. 24508898

[18] Kranz J, Schlager D, Mühlstädt S, Nagler J, Wagenlehner FME, Schneidewind L. 2019. Barriers to guideline adherence: identification of barriers to guideline adherence using a survey on the AWMF S3 guideline epidemiology, diagnosis, treatment, and management of uncomplicated bacterial, community-acquired urinary tract infections in adult patients. Urologe A 58:1019–1028. doi:10.1007/s00120-018-0848-3. 30623216

[19] Ironmonger D, Edeghere O, Verlander NQ, Gossain S, Hopkins S, Hilton B, Hawkey PM. 2018. Effect of general practice characteristics and antibiotic prescribing on *Escherichia coli* antibiotic non-susceptibility in the West Midlands region of England: a 4 year ecological study. J Antimicrob Chemother 73:787–794. doi:10.1093/jac/dkx465. 29309593

[20] Mulder M, Kiefte-de Jong JC, Goessens WHF, der Visser H, Hofman A, Stricker BH, Verbon A. 2017. Risk factors for resistance to ciprofloxacin in community-acquired urinary tract infections due to *Escherichia coli* in an elderly population. J Antimicrob Chemother 72:281–289. doi:10.1093/jac/dkw399. 27655855

[21] Anesi JA, Lautenbach E, Nachamkin I, Garrigan C, Bilker WB, Omorogbe J, Dankwa L, Wheeler M, Tolomeo P, Han JH, the CDC Prevention Epicenters Program. 2019. The role of extended-spectrum cephalosporin-resistance in recurrent community-onset Enterobacteriaceae urinary tract infections: a retrospective cohort study. BMC Infect Dis 19:163. doi:10.1186/s12879-019-3804-y. 30764770 PMC6376680

[22] Fasugba O, Gardner A, Mitchell BG, Mnatzaganian G. 2015. Ciprofloxacin resistance in community- and hospital-acquired Escherichia coli urinary tract infections: a systematic review and meta-analysis of observational studies. BMC Infect Dis 15:545. doi:10.1186/s12879-015-1282-4. 26607324 PMC4660780

[23] Kim HY, Lee SJ, Lee DS, Yo JM, Choe H. 2016. Microbiological characteristics of unresolved acute uncomplicated cystitis. Microb Drug Resist 22:387–391. doi:10.1089/mdr.2015.0241. 26780182

[24] Cuevas O, Cercenado E, Gimeno M, Marin M, Coronel P, Bouza E, Spanish Urinary Tract Infection Study Group (SUTIS). 2010. Comparative in vitro activity of cefditoren and other antimicrobials against Enterobacteriaceae causing community-acquired uncomplicated urinary tract infections in women: a Spanish nationwide multicenter study. Diagn Microbiol Infect Dis 67:251–260. doi:10.1016/j.diagmicrobio.2010.02.013. 20542206

[25] Delisle G, Quach C, Domingo M, Boudreault AA, Gourdeau M, Bernatchez H, Lavallee C. 2016. *Escherichia coli* antimicrobial susceptibility profile and cumulative antibiogram to guide empirical treatment of uncomplicated urinary tract infection in women in the province of Quebec, 2010–15. J Antimicrob Chemother 71:3562–3567. doi:10.1093/jac/dkw302. 27494927

[26] Schito GC, Naber KG, Botto H, Palou J, Mazzei T, Gualco L, Marchese A. 2009. The ARESC study: an international survey on the antimicrobial resistance of pathogens involved in uncomplicated urinary tract infections. Int J Antimicrob Agents 34:407–413. doi:10.1016/j.ijantimicag.2009.04.012. 19505803

[27] Kahlmeter G, Poulsen HO. 2012. Antimicrobial susceptibility of Escherichia coli from community-acquired urinary tract infections in Europe: the ECO·SENS study revisited. Int J Antimcrob Agents 39:45–51. doi:10.1016/j.ijantimicag.2011.09.013. 22055529

[28] Kahlmeter G, Åhman J, Matuschek E. 2015. Antimicrobial resistance of Escherichia coli causing uncomplicated urinary tract infections: a European update for 2014 and comparison with 2000 and 2008. Infect Dis Ther 4:417–423. doi:10.1007/s40121-015-0095-5. 26507395 PMC4675763

[29] Schmiemann G, Gágyor I, Hummers-Pradier E, Bleidorn J. 2012. Resistance profiles of urinary tract infections in general practice—an observational study. BMC Urol 12:33. doi:10.1186/1471-2490-12-33. 23171154 PMC3534546

[30] Seitz M, Stief C, Waidelich R. 2017. Local epidemiology and resistance profiles in acute uncomplicated cystitis (AUC) in women: a prospective cohort study in an urban urological ambulatory setting. BMC Infect Dis 17:685. doi:10.1186/s12879-017-2789-7. 29037164 PMC5644167

[31] van Driel AA, Notermans DW, Meima A, Mulder M, Donker GA, Stobberingh EE, Verbon A. 2019. Antibiotic resistance of Escherichia coli isolated from uncomplicated UTI in general practice patients over a 10-year period. Eur J Clin Microbiol Infect Dis 38:2151–2158. doi:10.1007/s10096-019-03655-3. 31440915 PMC6800841

[32] Kornfält Isberg H, Melander E, Hedin K, Mölstad S, Beckman A. 2019. Uncomplicated urinary tract infections in Swedish primary care; etiology, resistance and treatment. BMC Infect Dis 19:155. doi:10.1186/s12879-019-3785-x. 30760219 PMC6375206

[33] Stefaniuk E, Suchocka U, Bosacka K, Hryniewicz W. 2016. Etiology and antibiotic susceptibility of bacterial pathogens responsible for community-acquired urinary tract infections in Poland. Eur J Clin Microbiol Infect Dis 35:1363–1369. doi:10.1007/s10096-016-2673-1. 27189078 PMC4947106

[34] Heytens S, Boelens J, Claeys G, DeSutter A, Christiaens T. 2017. Uropathogen distribution and antimicrobial susceptibility in uncomplicated cystitis in Belgium, a high antibiotic prescribing country 20-year surveillance. Eur J Clin Microbiol Infect Dis 36:105–113. doi:10.1007/s10096-016-2776-8. 27639858

[35] Ismail MD, Ali I, Hatt S, Salzman EA, Cronenwett AW, Marrs CF, Rickard AH, Foxman B. 2018. Association of *Escherichia coli* ST131 lineage with risk of urinary tract recurrence among young women. J Glob Antimicrob Resist 13:81–84. doi:10.1016/j.jgar.2017.12.006. 29258889

[36] Filiatrault L, McKay RM, Patrick DM, Roscoe DL, Quan G, Brubacher J, Collins KM. 2012. Antibiotic resistance in isolates recovered from women with community-acquired urinary tract infections presenting to a tertiary care emergency department. CJEM 14:295–305. doi:10.2310/8000.2012.120666. 22967697

[37] Peyko V, Daves A, Eggleston M. 2019. Comparing an emergency department-specific antibiogram versus hospital-wide antibiogram and therapeutic dilemmas for uncomplicated cystitis. Infect Dis Clin Pract 27:155–159. doi:10.1097/IPC.0000000000000721.

[38] Olson RP, Harrell LJ, Kaye KS. 2009. Antibiotic resistance in urinary isolates of *Escherichia coli* from college women with urinary tract infections. Antimicrob Agents Chemother 53:1285–1286. doi:10.1128/AAC.01188-08. 19104022 PMC2650563

[39] Matsumoto T, Hamasuna R, Ishikawa K, Takahashi S, Yasuda M, Hayami H, Tanaka K, Kiyota H, Muratani T, Monden K, Arakawa S, Yamamoto S. 2011. Nationwide survey of antibacterial activity against clinical isolates from urinary tract infections in Japan (2008). Int J Antimicrob Agents 37:210–218. doi:10.1016/j.ijantimicag.2010.10.032. 21242062

[40] Shigemura K, Yamashita M, Shigemura K, Tanaka K, Arakawa S, Fujisawa M, Adachi M, Shigemura K. 2011. Chronological change of antibiotic use and antibiotic resistance in Escherichia coli causing urinary tract infections. J Infect Chemother 17:646–651. doi:10.1007/s10156-011-0241-2. 21487942

[41] Etani T, Naiki T, Yamaguchi S, Mori S, Nagai T, Iida K, Ando R, Kawai N, Tozawa K, Mogami T, Yasui T. 2017. Antimicrobial susceptibility of pathogens in acute uncomplicated cystitis cases in the urology department of a community hospital in Japan: comparison with treatment outcome and hospital-wide antibiogram. J Infect Chemother 23:692–697. doi:10.1016/j.jiac.2017.07.011. 28807755

[42] Lee G, Cho Y, Shim BS, Lee SD. 2010. Risk factors for antimicrobial resistance among the Escherichia coli strains isolated from Korean patients with acute uncomplicated cystitis: a prospective and nationwide study. J Korean Med Sci 25:1205–1209. doi:10.3346/jkms.2010.25.8.1205. 20676334 PMC2908792

[43] Wong CKM, Kung K, Au-Doung PLW, Ip M, Lee N, Fung A, Wong SYS. 2017. Antibiotic resistance rates and physician antibiotic prescription patterns of uncomplicated urinary tract infections in southern Chinese primary care. PLoS One 12:e0177266. doi:10.1371/journal.pone.0177266. 28486532 PMC5423680

[44] Ho PL, Yip KS, Chow KH, Lo JYC, Que T, Yuen K. 2010. Antimicrobial resistance among uropathogens that cause acute uncomplicated cystitis in women in Hong Kong: a prospective multicentre study in 2006 to 2008. Diagn Microbiol Infect Dis 66:87–93. doi:10.1016/j.diagmicrobio.2009.03.027. 19446980

[45] Jadoon RJ, Jalal-Ud-Din M, Khan SA. 2015. *E. coli* resistance to ciprofloxacin and common associated factors. J Coll Physicians Surg Pak 25:824–827. 26577970

[46] Aypak C, Altunsoy A, Düzgün N. 2009. Empiric antibiotic therapy in acute uncomplicated urinary tract infections and fluoroquinolone resistance: a prospective observational study. Ann Clin Microbiol Antimicrob 8:27. doi:10.1186/1476-0711-8-27. 19852849 PMC2770515

[47] Azap ÖK, Arslan H, Şerefhanoğlu K, Çolakoğlu Ş, Erdoğan H, Timurkaynak F, Senger SS. 2010. Risk factors for extended-spectrum beta-lactamase positivity in uropathogenic Escherichia coli isolated from community-acquired urinary tract infections. Clin Microbiol Infect 16:147–151. doi:10.1111/j.1469-0691.2009.02941.x. 19689464

[48] Al-Zahrani J, Al Dossari K, Gabr AH, Ahmed A-F, Al Shahrani SA, Al-Ghamdi S. 2019. Antimicrobial resistance patterns of uropathogens isolated from adult women with acute uncomplicated cystitis. BMC Microbiol 19:237. doi:10.1186/s12866-019-1612-6. 31666014 PMC6822473

[49] Kudinha T, Johnson JR, Andrew SD, Kong F, Anderson P, Gilbert GL. 2013. *Escherichia coli* sequence type 131 as a prominent cause of antibiotic resistance among urinary *Escherichia coli* isolates from reproductive-age women. J Clin Microbiol 51:3270–3276. doi:10.1128/JCM.01315-13. 23885001 PMC3811657

[50] Miotla P, Romanek-Piva K, Bogusiewicz M, Markut-Miotla E, Adamiak A, Wrobel A, Zebrowska M, Wawrysiuk S, Mendyk K, Rechberger E, Jakubczak A, Rechberger T. 2017. Antimicrobial resistance patterns in women with positive urine cultures: does menopausal status make a difference? Biomed Res Int 2017:4192908. doi:10.1155/2017/4192908. 28497048 PMC5406742

[51] Matsumoto T, Hamasuna R, Ishikawa K, Takahashi S, Yasuda M, Hayami H, Tanaka K, Muratani T, Monden K, Arakawa S, Yamamoto S. 2012. Sensitivities of major causative organisms isolated from patients with acute uncomplicated cystitis against various antibacterial agents: results of subanalysis based on the presence of menopause. J Infect Chemother 18:597–607. doi:10.1007/s10156-012-0419-2. 22572853

[52] Zhang X, Cui Y, Liu C, Zuo K, Tang Y. 2019. Antibiotic sales in primary care in Hubei province, China: an analysis of 2012–2017 procurement records. Int J Environ Res Public Health 16:3376. doi:10.3390/ijerph16183376. PMC6765864 31547325

[53] Kim B, Kim Y, Hwang H, Kim J, Kim S, Bae I, Choi WS, Jung SI, Jeong HW, Pai H. 2018. Trends and correlation between antibiotic usage and resistance pattern among hospitalized patients at university hospitals in Korea, 2004 to 2012. A Nationwide Multicenter Study. Medicine 97:e13719. doi:10.1097/MD.0000000000013719. 30572507 PMC6320075

[54] Kim YA, Park YS, Youk T, Lee H, Lee K. 2018. Trends in South Korean antimicrobial use and association with changes in Escherichia coli resistance rates: 12-year ecological study using a nationwide surveillance and antimicrobial prescription database. PLoS One 13:e0209580. doi:10.1371/journal.pone.0209580. 30596704 PMC6312334

[55] Terahara F, Nishiura H. 2019. Fluoroquinolone consumption and *Escherichia coli* resistance in Japan: an ecological study. BMC Public Health 19:246. doi:10.1186/s12889-019-6804-3. 31014305 PMC6480435

[56] Yang P, Chen Y, Jiang S, Shen P, Lu X, Xiao Y. 2020. Association between the rate of fluoroquinolones-resistant gram-negative bacteria and antibiotic consumption from China based on 145 tertiary hospitals data in 2014. BMC Infect Dis 20:269. doi:10.1186/s12879-020-04981-0. 32264851 PMC7137221

[57] Percival KM, Valenti KM, Schmittling SE, Strader BD, Lopez RR, Bergman SJ. 2015. Impact of antimicrobial stewardship intervention on urinary tract infection treatment in the ED. Am J Emerg Med 33:1129–1133. doi:10.1016/j.ajem.2015.04.067. 26027885

[58] Shively NR, Buehrle DJ, Clancy CJ, Decker BK. 2018. Prevalence of inappropriate antibiotic prescribing in primary care clinics within a Veterans Affairs Health Care system. Antimicrob Agents Chemother 62:e00337-18. doi:10.1128/AAC.00337-18. 29967028 PMC6105840

[59] Gordon AS, Adamson WC, DeVries AR. 2017. Virtual visits for acute, nonurgent care: a claims analysis of episode-level utilization. J Med Internet Res 19:e35. doi:10.2196/jmir.6783. 28213342 PMC5336603

[60] Mehrotra A, Paone S, Martich GD, Albert SM, Shevchik GJ. 2013. A comparison of care at e-visits and physician office visits for sinusitis and urinary tract infection. JAMA Intern Med 173:72–74. doi:10.1001/2013.jamainternmed.305. 23403816 PMC3889474

[61] Auta A, Hadi MA, Oga E, Adewuyi EO, Abdu-Aguye SN, Adeloye D, Strickland-Hodge B, Morgan DJ. 2019. Global access to antibiotics without prescription in community pharmacies: a systematic review and analysis. J Infect 78:8–18. doi:10.1016/j.jinf.2018.07.001. 29981773

[62] World Health Organization Regional Office for Europe. 2019. Assessing non-prescription and inappropriate use of antibiotics. Report on survey. World Health Organization, Geneva, Switzerland. https://apps.who.int/iris/bitstream/handle/10665/312306/9789289054089-eng.pdf.

[63] Lescure D, Paget J, Schellevis F, van Dijk L. 2018. Determinants of self-medication with antibiotics in European and Anglo-Saxon countries: a systematic review of the literature. Front Public Health 6:370. doi:10.3389/fpubh.2018.00370. 30619809 PMC6304439

[64] Zoorob R, Grigoryan L, Nash S, Trautner BW. 2016. Nonprescription antimicrobial use in a primary care population in the United States. Antimicrob Agents Chemother 60:5527–5532. doi:10.1128/AAC.00528-16. 27401572 PMC4997852

[65] Anger J, Lee U, Ackerman AL. 2019. Recurrent uncomplicated urinary tract infections in women: AUA/CUA/SUFU guideline (2019). American Urological Association, Linthicum Heights, MD. https://www.auanet.org/guidelines/recurrent-uti.

[66] Gupta K, Hooton TM, Roberts PL, Stamm WE. 2001. Patient-initiated treatment of uncomplicated recurrent urinary tract infections in young women. Ann Intern Med 135:9–16. doi:10.7326/0003-4819-135-1-200107030-00004. 11434727

[67] Córdoba G, Holm A, Hansen F, Hammerum AM, Bjerrum L. 2017. Prevalence of antimicrobial resistant *Escherichia coli* from patients with suspected urinary tract infection in primary care, Denmark. BMC Infect Dis 17:670. doi:10.1186/s12879-017-2785-y. 29017466 PMC5635483

[68] Shamseer L, Moher D, Clarke M, Ghersi D, Liberati A, Petticrew M, Shekelle P, Stewart LA, the PRISMA-P Group. 2015. Preferred reporting items for systematic review and meta-analysis protocols (PRISMA-P) 2015: elaboration and explanation. BMJ 350:g7647. doi:10.1136/bmj.g7647. 25555855

[69] Downes MJ, Brennan ML, Williams HC, Dean RS. 2016. Development of a critical appraisal tool to assess the quality of cross-sectional studies (AXIS). BMJ Open 6:e011458. doi:10.1136/bmjopen-2016-011458. PMC5168618 27932337

[70] Etienne M, Lefebvre E, Frebourg N, Hamel H, Pestel-Caron M, Caron F, Bacyst Study Group. 2014. Antibiotic treatment of acute uncomplicated cystitis based on rapid urine test and local epidemiology: lessons from a primary care series. BMC Infect Dis 14:137. doi:10.1186/1471-2334-14-137. 24612927 PMC3975248

[71] Katsarolis I, Poulakou G, Athanasia S, Kourea-Kremastinou J, Lambri N, Karaiskos E, Panagopoulos P, Kontopidou FV, Voutsinas D, Koratzanis G, Kanellopoulou M, Adamis G, Vagiakou H, Perdikaki P, Giamarellou H, Kanellakopoulou K, Collaborative Study Group on Antibiotic Resistance in Community-acquired Urinary Tract Infections. 2010. Acute uncomplicated cystitis: from surveillance data to a rationale for empirical treatment. Int J Antimicrob Agents 35:62–67. doi:10.1016/j.ijantimicag.2009.08.018. 19906513

[72] Hatzaki D, Poulakou G, Katsarolis I, Lambri N, Souli M, Deliolanis I, Nikolopoulos GK, Lebessi E, Giamarellou H. 2012. Cefditoren: comparative efficacy with other antimicrobials and risk factors for resistance in clinical isolates causing UTIs in outpatients. BMC Infect Dis 12:228. doi:10.1186/1471-2334-12-228. 23009290 PMC3518207

[73] Plate A, Kronenberg A, Risch M, Mueller Y, Di Gangi S, Rosemann T, Senn O. 2019. Active surveillance of antibiotic resistance patterns in urinary tract infections in primary care in Switzerland. Infection 47:1027–1035. doi:10.1007/s15010-019-01361-y. 31595436

[74] Gangcuangco LM, Alejandria M, Evans Henson K, Alfaraz L, Ata RM, Lopez M, Saniel M. 2015. Prevalence and risk factors for trimethoprim-sulfamethoxazole-resistant *Escherichia coli* among women with acute uncomplicated urinary tract infection in a developing country. Int J Infect Dis 34:55–60. doi:10.1016/j.ijid.2015.02.022. 25748571

