# Supplementary material for: Escherichia coli Resistance to Fluoroquinolones in Community-Acquired Uncomplicated Urinary Tract Infection in Women: a Systematic Review
Source: Antimicrob Agents Chemother. 2020 Sep 21;64(10):e00862-20. doi: 10.1128/AAC.00862-20 (PMC7508571; doi:10.1128/AAC.00862-20)
Supplement: Supplemental file 1 — Supplemental material. Download aac.00862-20-s0001.pdf, PDF file [file aac.00862-20-s0001.pdf]

Supplement to *E. coli* Resistance to Fluoroquinolones in Community-acquired Uncomplicated Urinary Tract Infection in Women: A Systematic Review

Ann E. Stapleton, Florian M.E. Wagenlehner, Aruni Mulgirigama, Monique Twynholm

APPENDIX 1. Search strategies

Embase

The PICO search function in EMBASE was used and included exploded terms for the keywords below from Emtree with their synonyms, including drug trade names. Keywords were also entered as free text terms.

urinary tract infection OR UTI OR cystitis OR acute cystitis OR uncomplicated urinary tract infection OR uncomplicated cystitis

AND

fluoroquinolone OR quinolone OR ciprofloxacin OR gemifloxacin OR levofloxacin OR norfloxacin OR ofloxacin OR prufloxacin OR tosufloxacin OR pefloxacin

AND

antibiotic resistance OR antimicrobial resistance OR fluoroquinolone resistance OR multidrug resistance

AND

escherichia coli OR e. coli

Filters:

Age (selected: adolescent; young adult; adult; middle aged; aged; very elderly)

Gender: female

Date of publication: January 1, 2009 to December 2, 2019

PubMed

#	Searches
1	urinary tract infection OR UTI OR cystitis OR acute cystitis OR uncomplicated urinary tract infection OR uncomplicated UTI OR uncomplicated cystitis OR ‘urinary tract infections’ (MeSH)
2	antimicrobial resist* OR antibiotic resist* OR drug resist* OR bacterial resist* OR multidrug resist* OR “drug resistance, microbial” (MeSH) OR “drug resistance, bacterial” (MeSH)

3 fluoroquinolone OR quinolone OR ciprofloxacin OR gemifloxacin OR levofloxacin OR norfloxacin OR ofloxacin OR prufloxacin OR tosufloxacin OR pefloxacin

4 Escherichia coli OR E. coli OR “Escherichia coli” (MeSH)

5 Date – publication: 2009/01/01 to present

1 AND 2 AND 3 AND 4 AND 5

TABLE S1 Characteristics of included studies according to geographical region:[a] fluoroquinolone resistance in females with community-acquired uncomplicated urinary tract infection caused by *E. coli*

**Country**	**Setting**	**Study design**	**Study period**	**Age**	**Culture-positive uUTI, n**	**uUTI caused by *E. coli*, %**	***E. coli* resistance data available, n**	**Susceptibility testing**	***E. coli* isolate susceptible to FQ, %**	***E. coli* isolates resistant to FQ, %**
**Europe**										
9 European countries and Brazil (1)	PC/ OPD	PRO	2003 to 2006	18-65 years	3181	76.7	Non-recurrent: 2062; recurrent: 253	CLSI	<u>CIP</u><br>Non-recurrent: 92.8; recurrent: 83.0	<u>CIP</u><br>Non-recurrent: 7.0; recurrent: 17.0
Austria (2)	PC/ OPD	PRO	June 2007 to Nov 2008	18-65 years	146	100	146	SRGA	NR	CIP: 4.1
Belgium (3)	PC	PRO	May 2014 to Dec 2015	>18 years	212	81.6	173	EUCAST	<u>LEV/OFL</u><br>18-55 years: 95.8; >55 years: 90.6	NR
Denmark (4)	PC	PRO	Dec 2014 to Dec 2015	18-65 years	105	69	105	EUCAST	NR	CIP: 8
France (1)	PC/ OPD	PRO	2003 to 2006	18-65 years	488	83.8	409	CLSI	CIP: 98.4	CIP: 1.4
France (5)	PC	PRO	2009 to 2011	18-65 years	199	77	157	CASFM	LEV: 97; OFL: 97	NR
France (6)	PC	PRO	2014	18-65 years	166	100	166	EUCAST	CIP: 95.2	NR

Country	Setting	Study design	Study period	Age	Culture-positive uUTI, n	uUTI caused by *E. coli*, %	*E. coli* resistance data available, n	Susceptibility testing	*E. coli* isolate susceptible to FQ, %	*E. coli* isolates resistant to FQ, %
Germany (7)	PC	PRO	Fall 2011	≥18 years	191	72.8	139	DIN, EUCAST, CLSI	CIP: 91.3	CIP: 8.7
Germany (8)	OPD	PRO	Jan 2015 to Jan 2017	≥18 years	423	86.3	365	EUCAST	CIP: 84.9; LEV: 86.3; MOX: 86.0	NR
Greece (9)	OPD	PRO	Jan 2005 to March 2006	>16 years	889	85.4	NR	CLSI	NR	CIP: 2.2
Greece (10)	OPD	PRO	Jan 2005 to Mar 2007	≥16 years	172	100	119	CLSI	NR	CIP: 1.7
Greece (2)	PC/ OPD	PRO	June 2007 to Nov 2008	18-65 years	209	100	209	SRGA	NR	CIP: 5.7
Netherlands (11)	PC	PRO	Jan 2009 to July 2009	≥11 years	785	72	489	EUCAST	CIP<br>All patients: 97; 11-20 years: 98; 21-50 years: 99; 51-70 years: 95, >70 years: 97<br>NOR<br>All patients: 97; 11-20 years: 97; 21-50 years: 98; 51-70	NR

Country	Setting	Study design	Study period	Age	Culture-positive uUTI, n	uUTI caused by *E. coli*, %	*E. coli* resistance data available, n	Susceptibility testing	*E. coli* isolate susceptible to FQ, %	*E. coli* isolates resistant to FQ, %
									years: 95; >70 years: 97	
Netherlands (12)	PC	PRO	Jan 2014 to Jan 2015	≥11 years	535	83	445	EUCAST	CIP<br>All patients: 94; 11-20 years: 93; 21-50 years: 96; 51-70 years: 97; >70 years: 88	NR
Poland (13)	OPD	PRO	Mar to May 2013	19-94 years	144	<65 years: 86.7: ≥65 years: 72.1	116	EUCAST	CIP<br>All patients: 75.9; <65 years: 88.9; ≥65 years: 54.6	NR
Poland (14)	OPD	RET	2013 to 2015	Adults	PRE: 2748; POST: 1705	PRE: 63.0; POST: 69.6	NR	NR	NR	CIP<br>PRE: 10.7; POST: 22.7
Portugal (2)	PC/ OPD	PRO	June 2007 to Nov 2008	18-65 years	144	100	144	SRGA	NR	CIP: 7.6

Country	Setting	Study design	Study period	Age	Culture-positive uUTI, n	uUTI caused by *E. coli*, %	*E. coli* resistance data available, n	Susceptibility testing	*E. coli* isolate susceptible to FQ, %	*E. coli* isolates resistant to FQ, %
Spain (15)	OPD	PRO	June 2008 to Mar 2009	Adults	2152	82	1761	CLSI	NR	CIP: 22.9; LEV: 22.5; NOR: 23.3
Spain (6)	PC	PRO	2014	18-65 years	169	100	169	EUCAST	CIP: 69.2	CIP: 30.8
Sweden (2)	PC/ OPD	PRO	June 2007 to Nov 2008	18-65 years	203	100	203	SRGA	NR	CIP: 2.5
Sweden (6)	PC	PRO	2014	18-65 years	137	100	137	EUCAST	CIP: 92.7	CIP: 7.3
Sweden (16)	PC	PRO	Nov 2014 to Mar 2016	≥17 years	243	72	176	EUCAST	NR	CIP: 1.1
Switzerland (17)	PC	PRO	Jun 2017 to Aug 2018	≥18 years	1055	74.8	695	ANRESIS	CIP: 89.1; LEV: 86.5	NR
UK (2)	PC/ OPD	PRO	June 2007 to Nov 2008	18-65 years	201	100	201	SRGA	NR	CIP: 0.5
UK (6)	PC	PRO	2014	18-65 years	124	100	124	EUCAST	CIP: 84.7	CIP: 15.3
**North America**										
Canada (18)	ED	PRO	2008	≥19 years	196	85.7	168	CLSI	CIP: 90.5	CIP: 8.9
Canada (19)	PC	PRO	Apr 2009 to Mar 2011	≥16 years	263	79.1	113	CLSI	NR	CIP: 4.4
Canada (20)	ED/ OPD	RET	Apr 2010 to Mar 2015	18-65 years	69,684	100	36,293	CLSI	CIP<br>2010: 92.1;<br>2011: 92.5;<br>2012: 91.4;	NR

Country	Setting	Study design	Study period	Age	Culture-positive uUTI, n	uUTI caused by *E. coli*, %	*E. coli* resistance data available, n	Susceptibility testing	*E. coli* isolate susceptible to FQ, %	*E. coli* isolates resistant to FQ, %
									2013: 90.7;<br>2014: 90.3	
US (21)	PC	RET	Jan 2005 to Dec 2007	College students	176	100	176	NR	NR	CIP: 6.8
US (22)	OPD	PRO	2005 to 2007	18-40 years	221	100	221	CLSI	NR	CIP: 2.8;<br>LEV: 2.8
US (23)	ED	RET	Sep 2016 to Feb 2017	Age ≥14 years	258	67.8	128	CLSI	LEV: 88	NR
**South America**										
Brazil (24)	PC/OPD	RET	Jan 2007 to Jan 2009	Age ≥14 years	NR	64.7	622	CLSI	NR	CIP: 9.2;<br>LEV: 18.0;<br>NOR: 7.4
**Asia Pacific**										
Australia (25)	PC/OPD	PRO	Jun 2009 to Jul 2011	15-45 years	322	100	322	CLSI	NR	CIP: 13;<br>NOR: 12
China (26)	PC	PRO	Jan 2012 to Dec 2013	≥16 years	141	75.9	107	CLSI	CIP: 76.6	CIP: 23.4
Hong Kong (27)	PC/ED/OPD	PRO	Jan 2006 to June 2008	≥18 years	352	77	271	CLSI	CIP: 87.1	CIP: 12.9
Japan (28)	OPD	PRO	Jan to Dec 2008	Adults	1009	63.3	831	CLSI	CIP: 91.8;<br>LEV: 91.8;<br>SIT: 98.0;<br>TOS: 91.5	NR

Country	Setting	Study design	Study period	Age	Culture-positive uUTI, n	uUTI caused by *E. coli*, %	*E. coli* resistance data available, n	Susceptibility testing	*E. coli* isolate susceptible to FQ, %	*E. coli* isolates resistant to FQ, %
Japan (29)	OPD	PRO	Jan to Dec 2008	Adults	PRE: 489; POST: 501	PRE: 65.0; POST: 61.5	PRE: 412; POST: 404	CLSI	CIP<br>PRE: 94.4; POST: 89.4<br>LEV<br>PRE: 94.4; POST: 89.5<br>SIT<br>PRE: 99.3; POST: 96.5<br>TOS<br>PRE: 93.9; POST: 89.1	NR
Japan (30)	OPD	RET	2007 to 2009		423	100	NR	CLSI	LEV: 85.8	NR
Japan (31)	OPD	RET	Aug 2015 to May 2017	16-90 years	165	74.4	123	CLSI	LEV<br>All ages: 84.6; <65 years: 83.3; 65 years: 85.2	LEV<br>All ages: 15.4; <65 years: 16.7; 65 years: 14.8
Korea (32)	OPD	PRO	May to Oct 2006	18-65 years	225	100	221	NCCLS (now CLSI)	NR	CIP: 26.7
Korea (33)	OPD	PRO	Jan 2008 to June 2009	Adults	1474	72.7	1071	CLSI	NR	CIP: 24.8<br>LEV: 21.3

Country	Setting	Study design	Study period	Age	Culture-positive uUTI, n	uUTI caused by *E. coli*, %	*E. coli* resistance data available, n	Susceptibility testing	*E. coli* isolate susceptible to FQ, %	*E. coli* isolates resistant to FQ, %
Korea (34)	OPD	RET	Jan 2010 to Dec 2014	Adults	259	100	259	CLSI	CIP: 58.3; LEV: 61.3	NR
Pakistan (35)	OPD	PRO	Dec 2011 to June 2012	≥18 years	125	100	125	CLSI	CIP<br>All patients: 67.2; 18-30 years: 89.7; 31-40 years: 64.3; 41-50 years: 58.1; 51-60 years: 58.1; 61-75 years: 61.5	CIP<br>All patients: 32.8; 18-30 years: 10.3; 31-40 years: 35.7; 41-50 years: 41.9; 51-60 years: 41.9; 61-75 years: 38.5
Philippines (36)	ED/ OPD	PRO	July 2010 to Oct 2011	≥18 years	229	76.2	179	CLSI	NR	LEV: 5.7
Turkey (37)	ED/ OPD	PRO	Mar 2005 to Sep 2006	18-65 years	150	71.3	139	CLSI	NR	All FQs<br>All patients: 25.2; <50 years: 22.7; ≥50 years: 31.3
Turkey (38)	OPD	PRO	Jan to Dec 2007	18-65 years	289	93	269	CLSI	NR	CIP: 22
**Middle East**										
Saudi Arabia (39)	PC/ OPD	PRO	May 2015 to Apr 2016	≥18 years	179	70.4	126	NR	NR	CIP: 25.4

[a]Where available, data for individual countries from the same study are listed separately. Note: ECO·SENS 2014 update data from Germany have not been included as it could not be confirmed that data were exclusively from community-acquired uUTIs. Data from some individual European countries in the ARESC study have not been included where fewer than 100 *E. coli* isolates were analyzed. ARESC, Antimicrobial Resistance Epidemiological Survey on Cystitis; ANRESIS, Swiss Center for Antibiotic Resistances; CASFM, Comité de l'antibiogramme de la Société Française de Microbiologie; CIP, ciprofloxacin; CLSI, Clinical and Laboratory Standards Institute; DIN, Deutsches Institut für Normung; ED, emergency department; EUCAST, European Committee on Antimicrobial Susceptibility Testing; FQ, fluoroquinolones; LEV, levofloxacin; NCCLS, National Committee for Clinical Laboratory Standards; NOR, norfloxacin; NR, not reported; OFL, ofloxacin; OPD, outpatients department; PC, primary care; PRE, premenopausual; POST, postmenopausal; PRO, prospective; RET, retrospective; SIT: sitafloxacin; SRGA, Swedish Reference Group for Antibiotics; TOS: tosufloxacin; uUTI, uncomplicated urinary tract infection.

TABLE S2 Quality of studies/study reporting according to abbreviated AXIS criteria (40)

**Country**	**Study design appropriate for the stated aims**	**Target population clearly defined[a]**	**Sample represented target population under investigation**	**Selection process likely to select subjects representative of target population under investigation**	**Basic data adequately described**	**Limitations of the study discussed**
Australia (25)	+	+	+	+	+	+
Belgium (3)	+	+	+	+	+	×
Brazil (24)	+	?	Single urban area	+	+	+
Canada (18)	+	+	Single urban area	+	+	+
Canada (19)	+	+	+	+	+	+
Canada (20)	+	?	+	+	+	+
China (Wong)	+	+	+	+	+	+
Denmark (4)	+	?	+	+	+	+
Europe and Brazil (1)	+	?	+	+	+	×

Europe (2)	+	+	+	+	+	✕
Europe (6)	+	+	+	+	+	✕
France (5)	+	+	+	+	+	✕
Germany (7)	+	?	+	+	+	+
Germany (8)	+	+	Single urban area	+	+	+
Greece (9)	+	+	+	+	+	✕
Greece (10)	+	+	+	+	+	+
Hong Kong (27)	+	+	+	+	+	✕
Japan (28)	+	+	+	+	+	✕
Japan (30)	+	?	Single urban area	+	+	✕
Japan (31)	+	+	Single urban area	+	+	+
Korea (32)	+	+	+	+	+	✕

Korea (33)	+	+	+	+	+	×
Korea (34)	+	?	+	+	+	+
The Netherlands (11)	+	+	+	+	+	×
The Netherlands (12)	+	+	+	+	+	+
Pakistan (35)	+	+	+	+	+	+
Philippines (36)	+	+	Middle to upper class patients only in a single urban area	+	+	+
Poland (13)	+	?	+	Recruitment process unclear	+	×
Poland (14)	+	?	+	+	+	×
Saudi Arabia (39)	+	?	Single urban area	+	+	+
Spain (15)	+	+	+	+	+	×
Sweden (16)	+	+	+	+	+	+

Switzerland (17)	+	+	+	+	+	+
Turkey (37)	+	+	Single urban area	+	+	+
Turkey (38)	+	+	+	+	+	✕
US (21)	+	?	College setting	+	+	✕
US (22)	+	+	Participants in an interventional clinical trial	Participants in an interventional clinical trial	+	+
US (23)	+	?	Single urban area	+	+	+

Full details of diagnostic criteria presented in Table S2. Green crosses indicate that criteria were fulfilled; orange crosses indicate that not all criteria were fulfilled; red crosses indicate that criteria were not fulfilled. AXIS, Appraisal Tool for Cross-sectional Studies; uUTI, uncomplicated urinary tract infection.

Table S3 Diagnostic criteria used in the selected studies

Study	uUTI stated but not defined	uUTI symptoms	Postmenopausal women included	Recurrent UTI included	Pregnancy permitted	Upper UTI excluded	Urinary catheter excluded	Comorbidities excluded	Physical/ anatomical urinary tract abnormalities excluded
Australia (25)		YES	NO		NO	YES		Diabetes, diarrhea	YES
Belgium (3)		YES			NO	YES		Diabetes, urologic, renal disorders, gynecologic disorders, immuno-suppression	
Brazil (24)	YES								
Canada (18)		YES			NO	YES	YES	Urologic disorders, urinary incontinence, diabetes, renal failure, liver failure, immuno-suppression, cancer	YES
Canada (19)	YES						YES	Diabetes, urologic disorders	YES
Canada (20)	YES								
China (26)	YES				NO			Immuno-suppression	YES
Denmark (4)		YES			NO	YES		Comorbidities stated but not defined	
9 European Countries and Brazil (1)		YES	NO	YES	YES	YES	YES		YES
Europe (2)		YES		NO	NO	YES	YES	Diabetic complications, immuno-suppression, CNS disorders, venereal diseases	YES

Study	uUTI stated but not defined	uUTI symptoms	Postmenopausal women included	Recurrent UTI included	Pregnancy permitted	Upper UTI excluded	Urinary catheter excluded	Comorbidities excluded	Physical/ anatomical urinary tract abnormalities excluded
Europe (6)		YES		NO	NO	YES	YES	Diabetic complications, immuno-suppression, CNS disorders, venereal diseases	YES
France (5)		YES		NO	NO	YES	YES	Urinary tract disorders, diabetes, immuno-suppression, cancer	
Germany (7)	YES			YES	NO		NO	Neurologic disease	
Germany (8)	YES	YES	YES	YES	YES				
Greece (9)	YES	YES			NO		YES	Diabetes, kidney stones	
Greece (10)		YES		YES	NO	YES	YES	Diabetes, kidney stones, vaginal symptoms	
Hong Kong (27)		YES				YES	YES	Urologic disorders	
Japan (28)		YES	YES	YES			YES	Diabetes, cancer, cerebro-vascular disease, immuno-suppression	YES
Japan (30)	YES					YES		Diabetes, Cancer	
Japan (31)	YES				NO		YES	Diabetes, cancer, cerebro-vascular disease, immuno-suppression, asymptomatic bacteriuria, urologic disease	YES

Study	uUTI stated but not defined	uUTI symptoms	Postmenopausal women included	Recurrent UTI included	Pregnancy permitted	Upper UTI excluded	Urinary catheter excluded	Comorbidities excluded	Physical/ anatomical urinary tract abnormalities excluded
Korea (32)	YES	YES		YES					
Korea (33)		YES				YES	YES	Diabetes, urologic disorders	
Korea (34)		YES					YES	Diabetes, renal disorders	YES
The Netherlands (11)		YES					YES	Diabetes, urologic disorders, immuno-suppression	
The Netherlands (12)		YES			NO	YES		Diabetes, urologic disorders, immuno-suppression	
Pakistan (35)	YES			YES	NO	YES	NO	Immuno-suppression, psychiatric disorders, connective tissue disorders	
Philippines (36)	YES	YES			NO			Diabetes, immuno-suppression	YES
Poland (13)	YES			NO				Urinary incontinence, diabetes, renal disorders	
Poland (14)	YES			NO					
Saudi Arabia (39)		YES		NO	NO		NO	Urologic disorders	YES
Spain (15)	YES								YES
Sweden (16)		YES				YES	YES		
Switzerland (17)	YES				NO	YES		Urologic disorders	YES
Turkey (37)		YES		NO	NO	YES	YES	Diabetes, immuno-suppression, urologic disorders	YES

Study	uUTI stated but not defined	uUTI symptoms	Postmenopausal women included	Recurrent UTI included	Pregnancy permitted	Upper UTI excluded	Urinary catheter excluded	Comorbidities excluded	Physical/ anatomical urinary tract abnormalities excluded
Turkey (38)	YES			NO	NO		YES	Urologic disorders, bladder cancer	YES
US (21)	YES							Renal disorders	YES
US (22)		YES			NO		YES	Diabetes, urologic disorders	
US (23)	YES				NO				

CNS, central nervous system; uUTI, uncomplicated urinary tract infection.

REFERENCES

1. Schito GC, Naber KG, Botto H, Palou J, Mazzei T, Gualco L, Marchese A. 2009. The ARESC study: an international survey on the antimicrobial resistance of pathogens involved in uncomplicated urinary tract infections. Int J Antimicrob Agents 34:407–413.

2. Kahlmeter G, Poulsen HO. 2012. Antimicrobial susceptibility of Escherichia coli from community-acquired urinary tract infections in Europe: the ECO·SENS study revisited. Int J Antimcrob Agents 39:45–51.

3. Heytens S, Boelens J, Claeys G, DeSutter A, Christiaens T. 2017. Uropathogen distribution and antimicrobial susceptibility in uncomplicated cystitis in Belgium, a high antibiotic prescribing country 20-year surveillance. Eur J Clin Microbiol Infect Dis 36:105–113.

4. Córdoba G, Holm A, Hansen F, Hammerum AM, Bjerrum L. 2017. Prevalence of antimicrobial resistant *Escherichia coli* from patients with suspected urinary tract infection in primary care, Denmark. BMC Infect Dis 17:670.

5. Etienne M, Lefebvre E, Frebourg N, Hamel H, Pestel-Caron M, Caron F, Bacyst Study Group. 2014. Antibiotic treatment of acute uncomplicated cystitis based on rapid urine test and local epidemiology: lessons from a primary care series. BMC Infect Dis 14:137.

6. Kahlmeter G, Åhman J, Matuschek E. 2015. Antimicrobial resistance of Escherichia coli causing uncomplicated urinary tract infections: a European update for 2014 and comparison with 2000 and 2008. Infect Dis Ther 4:417–423.

7. Schmiemann G, Gágyor I, Hummers-Pradier E, Bleidorn J. 2012. Resistance profiles of urinary tract infections in general practice—an observational study. BMC Urol 12:33.

8. Seitz M, Stief C, Waidelich R. 2017. Local epidemiology and resistance profiles in acute uncomplicated cystitis (AUC) in women: a prospective cohort study in an urban urological ambulatory setting. BMC Infect Dis 17:685.

9. Katsarolis I, Poulakou G, Athanasia S, Kourea-Kremastinou J, Lambri N, Karaiskos E, Panagopoulos P, Kontopidou FV, Voutsinas D, Koratzanis G, Kanellopoulou M, Adamis G, Vagiakou H, Perdikaki P, Giamarellou H, Kanellakopoulou K. 2010. Acute uncomplicated cystitis: from surveillance data to a rationale for empirical treatment. Int J Antimicrob Agents 35:62–67.

10. Hatzaki D, Poulakou G, Katsarolis I, Lambri N, Souli M, Deliolanis I, Nikolopoulos GK, Lebessi E, Giamarellou H. 2012. Cefditoren: comparative efficacy with other

antimicrobials and risk factors for resistance in clinical isolates causing UTIs in outpatients. BMC Infect Dis 12:228.

11. den Heijer CDJ, Donker GA, Maes J, Stobberingh EE. 2010. Antibiotic susceptibility of unselected uropathogenic *Escherichia coli* from female Dutch general practice patients: a comparison of two surveys with a 5 year interval. J Antimicrob Chemother 65:2128–2133.

12. van Driel AA, Notermans DW, Meima, Mulder M, Donker GA, Stobberingh EE, Verbon A. 2019. Antibiotic resistance of Escherichia coli isolated from uncomplicated UTI in general practice patients over a 10-year period. Eur J Clin Microbiol Infect Dis 38:2151–2158.

13. Stefaniuk E, Suchocka U, Bosacka K, Hryniewicz W. 2016. Etiology and antibiotic susceptibility of bacterial pathogens responsible for community-acquired urinary tract infections in Poland. Eur J Clin Microbiol Infect Dis 35:1363–1369.

14. Miotla P, Romanek-Piva K, Bogusiewicz M, Markut-Miotla E, Adamiak A, Wrobel A, Zebrowska M, Wawrysiuk S, Mendyk K, Rechberger E, Jakubczak A, Rechberger T. 2017. Antimicrobial resistance patterns in women with positive urine cultures: does menopausal status make a difference? Biomed Res Int Article ID 4192908.

15. Cuevas O, Cercenado E, Gimeno M, Marin M, Coronel P, Bouza E, Spanish Urinary Tract Infection Study Group (SUTIS). 2010. Comparative in vitro activity of cefditoren and other antimicrobials against Enterobacteriaceae causing community-acquired uncomplicated urinary tract infections in women: a Spanish nationwide multicenter study. Diagn Microbiol Infect Dis 67:251–260.

16. Kornfält Isberg H, Melander E, Hedin K, Mölstad S, Beckman A. 2019. Uncomplicated urinary tract infections in Swedish primary care; etiology, resistance and treatment. BMC Infect Dis 19:155.

17. Plate A, Kronenberg A, Risch M, Mueller Y, Di Gangi S, Rosemann T, Senn O. 2019. Active surveillance of antibiotic resistance patterns in urinary tract infections in primary care in Switzerland. Infection 47:1027–1035.

18. Filiatrault L, McKay RM, Patrick DM, Roscoe DL, Quan G, Brubacher J, Collins KM. 2012. Antibiotic resistance in isolates recovered from women with community-acquired urinary tract infections presenting to a tertiary care emergency department. CJEM 14:295–305.

19. McIsaac WJ, Moineddin R, Meaney C, Mazzulli T. 2013. Antibiotic-resistant *Escherichia coli* in women with acute cystitis in Canada. Can J Infect Dis Med Microbiol 24:143–149.

20. Delisle G, Quach C, Domingo M, Boudreault AA, Gourdeau M, Bernatchez H, Lavallee C. 2016. *Escherichia coli* antimicrobial susceptibility profile and cumulative antibiogram to guide empirical treatment of uncomplicated urinary tract infection in women in the province of Quebec, 2010-15. J Antimicrob Chemother 71:3562–3567.

21. Olson RP, Harrell LJ, Kaye KS. 2009. Antibiotic resistance in urinary isolates of *Escherichia coli* from college women with urinary tract infections. Antimicrob Agents Chemother 53:1285–1286.

22. Ismail MD, Ali I, Hatt S, Salzman EA, Cronenwett AW, Marrs CF, Rickard AH, Foxman B. 2018. Association of *Escherichia coli* ST131 lineage with risk of urinary tract recurrence among young women. J Glob Antimicrob Resist 13:81–84.

23. Peyko V, Daves A, Eggleston M. 2019. Comparing an emergency department-specific antibiogram versus hospital-wide antibiogram and therapeutic dilemmas for uncomplicated cystitis. Infect Dis Clin Pract 27:155–159.

24. Araújo SM, Mourão TC, Oliverita JL, Melo IFS, Araújo CAA, Araújo NAA, Melo MCA, Araújo SR, Daher EF. 2011. Antimicrobial resistance of uropathogens in women with acute uncomplicated cystitis from primary care settings. Int Urol Nephrol 43:461–466.

25. Kudinha T, Johnson JR, Andrew SD, Kong F, Anderson P, Gilbert GL. 2013. *Escherichia coli* sequence type 131 as a prominent cause of antibiotic resistance among urinary *Escherichia coli* isolates from reproductive-age women. J Clin Microbiol 51:3270–3276.

26. Wong CKM, Kung K, Au-Duong PLW, Ip M, Lee N, Fung A, Wong SYS. 2017. Antibiotic resistance rates and physician antibiotic prescription patterns of uncomplicated urinary tract infections in southern Chinese primary care. PLoS One 12:e0177266.

27. Ho PL, Yip KS, Chow KH, Lo JYC, Que T, Yuen K. 2010. Antimicrobial resistance among uropathogens that cause acute uncomplicated cystitis in women in Hong Kong: a prospective multicentre study in 2006 to 2008. Diagn Microbiol Infect Dis 66:87–93.

28. Matsumoto T, Hamasuna R, Ishikawa K, Takahashi S, Yasuda M, Hayami H, Tanaka K, Kiyota H, Muratani T, Monden K, Arakawa S, Yamamoto S. 2011. Nationwide survey of antibacterial activity against clinical isolates from urinary tract infections in Japan (2008). *Int J Antimicrob Agents* 37:210–218.

29. Matsumoto T, Hamasuna R, Ishikawa K, Takahasi S, Yasuda M, Hayami H, Tanaka K, Muratani T, Monden K, Arakawa S, Yamamoto S. 2012. Sensitivities of major causative organisms isolated from patients with acute uncomplicated cystitis against various antibacterial agents: results of subanalysis based on the presence of menopause. J Infect Chemother 18:597–607.

30. Shigemura K, Tanaka K, Adachi S, Yamashita M, Arakawa S, Fujisawa M. 2011. Chronological change of antibiotic use and antibiotic resistance in Escherichia coli causing urinary tract infections. J Infect Chemother 17:646–651.

31. Etani T, Naiki T, Yamaguchi S, Mori S, Nagai T, Iida K, Ando R, Kawai N, Tozawa K, Mogami T, Yasui T. 2017. Antimicrobial susceptibility of pathogens in acute uncomplicated cystitis cases in the urology department of a community hospital in Japan: comparison with treatment outcome and hospital-wide antibiogram. J Infect Chemother 23:692–697.

32. Lee G, Cho Y, Shim BS, Lee SD. 2010. Risk factors for antimicrobial resistance among the Escherichia coli strains isolated from Korean patients with acute uncomplicated cystitis: a prospective and nationwide study. J Korean Med Sci 25: 1205–1209.

33. Lee SJ, Lee DS, Choe HS, Shim BS, Kim CS, Kim ME, Cho Y. 2011. Antimicrobial resistance in community-acquired urinary tract infections: results from the Korean Antimicrobial Resistance Monitoring System. J Infect Chemother 17:440–446.

34. Kim HY, Lee SJ, Lee DS, Yo JM, Choe H. 2016. Microbiological characteristics of unresolved acute uncomplicated cystitis. Microb Drug Resist 22:387–391.

35. Jadoon RJ, Jalal-ud-din M, Khan SA. 2015. *E. coli* resistance to ciprofloxacin and common associated factors. J Coll Physicians Surg Pak 25:824–827.

36. Gangcuangco LM, Alejandria M, Evans Henson K, Alfaraz L, Ata RM, Lopez M, Saniel M. 2015. Prevalence and risk factors for trimethoprim-sulfamethoxazole-resistant *Escherichia coli* among women with acute uncomplicated urinary tract infection in a developing country. Int J Infect Dis 34:55–60.

37. Aypak C, Altunsoy A, Düzgün N. 2009. Empiric antibiotic therapy in acute uncomplicated urinary tract infections and fluoroquinolone resistance: a prospective observational study. Ann Clin Microbiol Antimicrob 8:27.

38. Azap OK, Arslan H, Serefhanoglu K, Colakoglu S, Erdogan H, Timurkaynak F, Senger SS. 2010. Risk factors for extended-spectrum beta-lactamase positivity in uropathogenic Escherichia coli isolated from community-acquired urinary tract infections. Clin Microbiol

Infect 16:147–51.

39. Al-Zahrani J, Al Dossari K, Gabr AH, Ahmed A, Abdulrahman Al Sharani S, Al-Ghamdi S. 2019. Antimicrobial resistance patterns of uropathogens isolated from adult women with acute uncomplicated cystitis. BMC Microbiol 19:237.

40. Downes MJ, Brennan ML, Williams HC, Dean RS. 2016. Development of a critical appraisal tool to assess the quality of cross-sectional studies (AXIS). BMJ *Open* 6:e011458.
